# The Institutional Library

**Published:** 1921-09-17

**Authors:** 


### THE INSTITUTIONAL LIBRARY.

**Radiographic Technique.** By T. Thorne Baker. (Constable & Co., Ltd. Price 15s. net.)

This book deals mainly with the photographic aspect of radiographic technique, and does so in a most complete manner. There can be no disputing that this side of radiography is very generally neglected by the radiologist, and receives much less attention than it merits from the operator. It is certain that in the majority of hospital *x*-ray departments a large proportion of failures are due entirely to faulty work in the dark-room, while the same factor is responsible for a general standard considerably below that which should be attained. The first chapter deals briefly with the essentials of an *x*-ray apparatus. More space should be devoted to the protection of the *x*-ray worker; the recommendations of the Röntgen Society (1915), which are quoted in the appendix, requiring considerable amplification in the light of present-day knowledge. The book deserves to achieve a widespread popularity, both with radiologists and operators.

**Mother and Child.** By Edward P. Davis, A.M., M.D. Fourth edition (revised). (London: J. B. Lippincott Co. Pp. 278. 33 Illustrations. Price 12s. 6d. net.)

This book, by an American obstetrician, is divided into two parts, the first of which is devoted to the nine months of pregnancy as they affect both mother and child, while the second, after a short account of the puerperium, is devoted to the career of the child from the cradle up to the time when he is old enough to be a constant source of danger to himself and of apprehension to everyone about him. The instruction given follows safe if conventional lines; and, whilst possessing no particular merits over a multitude of similar books, this volume is thoroughly readable and reliable. Containing a good deal that is not strictly necessary to the successful upbringing of a child, it is capable of enlarging considerably the intelligent mother's horizon and of imparting an added dignity and reasonableness to her conduct of the training of her child—the most important mission of a woman's life.

**Œdema and Nephritis** By Martin H. Fischer. Third edition. (John Wiley & Sons, New York, and Chapman & Hall, Ltd., London. 1921. Price 55s. net.)

The first edition of this work was published as long ago as 1910. It is a high tribute to the merit of so technical a work that it should have reached a third edition in ten years.

It is impossible within the compass of a review to attempt to discuss or criticise the enormous mass of experimental evidence dealing with the production of œdema and nephritis which is brought forward by the author. Naturally, the book is not one which will make a wide appeal to any except those interested in the problems of the physiology and pathology of water-absorption in the living organism, but for those who have not the time or the special knowledge to examine the experimental evidence the author has devoted a chapter in which his main conclusions are outlined. There can be no doubt, however, that the book will prove to be of great value to all who are interested in the wide field of research which it covers. The quality of the paper and the printing leave nothing to be desired.

**An Atlas of Normal Labour.** By G. Drummond Robinson, M.D. Wm. Heinemann. 1921. Price 25s. net.)

Dr. Drummond Robinson's cinematograph films of labour for teaching purposes excited much interest when they were shown (released, perhaps one ought to say) some little time ago. He now publishes a collection of diagrams, based upon selected photographs from his films, illustrating the gradual progress of normal labour from start to finish; and with these a number of the actual photographs themselves. It is doubtful whether these diagrams and photographs teach the student any more than he can learn from other sources; in any case, the number of the diagrams is unnecessarily large, as in many cases the difference between successive pictures is negligible. With every desire to give credit for the author's pioneer work in medical education, we fear this book is too expensive for the usually none too affluent medical student.

